# Reliability and Validity of the Motor Activity Log (MAL-30) Scale for Post-Stroke Patients in a Spanish Sample

**DOI:** 10.3390/ijerph192214964

**Published:** 2022-11-14

**Authors:** Mirian Santamaría-Peláez, Rocío Pardo-Hernández, Jerónimo J. González-Bernal, Raúl Soto-Cámara, Josefa González-Santos, Jessica Fernández-Solana

**Affiliations:** Department of Health Sciences, University of Burgos, 09001 Burgos, Spain

**Keywords:** motor activity log, MAL-30, functionality, upper limb, stroke

## Abstract

Background: The validation of assessment instruments is of great importance when they are applied in clinical and healthcare settings, since their safe and reliable use is essential for the application of appropriate and high-quality treatments. The motor activity log (MAL-30) is an instrument widely used by professionals in the clinic, which has been validated in different countries, languages and populations. The aim of this study was to determine the reliability and validity of the MAL-30 scale for post-stroke patients in a Spanish sample. Methods: For this purpose, internal consistency tests were carried out using Cronbach’s alpha, item–item and item–total correlations, and a half-and-half test for reliability. For the validation, criterion validity tests were performed using the Fugl-Meyer assessment scale as the gold standard, and the convergent validity tests were carried out by correlation with the action research arm test (ARAT), box and block test (BBT), functional independence measure (FIM)–functional assessment measure (FAM), Lawton and Brody index and stroke quality of life scale (ECVI-38). Results: The results showed good internal consistency, as well as a good criterion and convergent validity. Conclusions: The MAL-30 instrument can be considered a valid and reliable tool for assessing the quantity and quality of the use of the affected upper limb in the performance of the activities of daily living and the instrumental activities of daily living after stroke in a Spanish sample.

## 1. Introduction

Stroke is considered one of the main causes of disability in many Western countries, with 13.7 million new cases every year worldwide [1,2,3], in addition to being one of the leading causes of death worldwide [1]. In Spain, the annual incidence rate is 252 per 100,000 inhabitants according to INE (National Statistics Institute) data, representing, therefore, the leading cause of disability in adulthood and the third leading cause of death as of 2020 [4,5,6]. This disability has great impacts at all levels for the person and the family, including the social, economic and health levels [4,6]. Likewise, this chronic disability has a serious impact on the individual’s daily life, which includes motor impairment in the upper limbs in up to 60% of cases [2,7].

Approximately 80% of stroke patients survive the acute phase, and most of them recover their ability to walk. However, between 30% and 66% of them experience long-term alterations at the level of the affected upper limb [3]. Patients may present with various difficulties in performing their occupations due to a certain degree of impairment or alterations. This can be observed especially in patients who have suffered a left hemiparetic stroke, experiencing worse results in rehabilitation and/or greater dependence in activities of daily living (ADL), associated with the neglect of their affected side [8].

Hemiparesis is one of the main neurological deficits or impairments resulting from a stroke that involves a lack of muscle strength and dissociated movement of the affected hemibody [9,10]. It is also one of the main causes of alterations in the patient’s daily life and affects the quality of life (QoL) [11,12]. Likewise, hemineglect is another disorder associated with stroke, which can complicate hemiparesis and plays a very important role in the overall recovery, especially in regard to the affected upper limb’s motor function and the restoration of functionality [12,13,14]. Hemineglect is a disorder defined as the inability to respond to stimuli in the hemisphere contralateral to the lesion [14,15,16]. This disorder may reduce the person’s ability to attend, look, listen or perform movements in the middle of their environment. Therefore, these patients show a limited response in regaining the ability for the independent performance of ADLs, instrumental activities of daily living (IADLs) and social and/or leisure activities [8,16]. The associated lack of awareness of neglect can lead to poor functional outcomes and may become the primary source of long-term disability after stroke [15].

In order to adapt to the motor disability that these symptoms generate, patients develop compensatory behaviors characterized by a decrease in the use of their affected arm and an increased use of the opposite arm, despite, in many cases, the functional recovery of their affected arm [10,17,18]. This behavior of the non-use of the affected arm, even in the presence of functional capacity, corresponds to the “learned non-use” phenomenon, described as the learned non-use between the impairment and the functional capacity or actual use of the upper limb [10,19].

The lack of recovery of motor ability and functionality and the presence of learned non-use, from a clinical point of view, can significantly reduce the occupational performance of the patients [10,11]. The patient’s report of their capacity for the spontaneous use of the affected upper limb in ADLs should be associated with both its efficient use and the subjective perception of its effectiveness [9,20], but to ensure an awareness of this, it is necessary to obtain valid and reliable outcome measures that assess individual qualities with the objectives of implementing personalized programs and evaluating the effectiveness of the interventions applied [21,22].

There are several assessment instruments used to measure upper limb function and/or impairment after stroke, such as the Wolf motor function test (WMFT), Fugl-Meyer assessment (FMA), motor activity log (MAL) and motor assessment scale (MAS), functional independence measure (FIM)–functional assessment measure (FAM), box and block test (BBT) or Lawton and Brody index [10,22]. Some studies have shown that the motor activity log (MAL) scale correlates directly with both the ability to manipulate objects and the perceived ability to use the affected limb [20]. On the other hand, previous studies have revealed that the impaired motor functioning of the paretic upper limb can be quantified using the upper extremity FMA assessment (FMA-UE), correlated significantly with people with stroke and with the action research arm test (ARAT) and MAL scales [23]. However, it is important to add the measurement of QoL when dealing with patients who have just suffered a stroke, since it is intimately linked to physical and functional health. There are specific scales to measure the QoL of stroke patients, such as the stroke quality of life scale (ECVI-38) [24,25].

The MAL-30 measure has been validated in several countries [20,22,26,27,28,29,30] and has been translated into Spanish, but it was uniquely adapted to the Hispanic American population [20,31]. However, some studies that were carried out in Spain [32,33,34] used this version that was translated into Spanish but not validated for this population. Because of this, despite the fact that it is a scale that is currently being used in various studies, the MAL assessment instrument has not been validated for post-stroke patients in the Spanish population. Therefore, this research’s aim was to test the reliability and validity of MAL-30 for the quantitative and qualitative measurement of the use of the diseased upper limb among people after a stroke in the Spanish population.

## 2. Materials and Methods

### 2.1. Participants

The study had a cross-sectional design and was carried out in the cities of Burgos and Cordoba (Spain) with a sample of 223 persons who had previously experienced a stroke. The population was recruited at the time of discharge from the Neurology Service and Stroke Unit of both hospitals by means of consecutive sampling.

### 2.2. Procedure

Once the collaboration and confidentiality agreement documents had been signed at the centers participating in the study, the data collection necessary to carry out the research was begun. The research plan was positively evaluated by the ethics committee of the Burgos University Hospital (HUBU), the IR Approval Committee HUBU 2134/2019. Randomized, controlled and blinded sampling was performed. In the participating centers, the persons designated for the data collection proceeded to collect the data and sent them to the research team after a process of anonymization (from that moment on, they were always treated anonymously and as a whole). The patient recruitment, data collection and data analysis were carried out by people other than the research team. The data collection was carried out by means of a battery composed of several scales that included the evaluation of the patient’s functionality, autonomy and QoL.

The inclusion criteria included participants over 18 years of age with a diagnosis of residual hemiparesis due to stroke, those whose movement of the affected upper extremities were classified between stages II and IV of the Brunnstrom Scale, and those whose upper limb movements were classified between stages II and IV of the Brunnstrom Scale [35]. The criteria for exclusion included the presence of aphasia and cognitive impairment greater than 28 on the MOCA [36]. All the participants signed an informed consent form before starting the study.

Once the data were obtained, a matrix was created for their evaluation using the IBM SPSS version 25.0 (IBM-Inc., Chicago, IL, USA).

### 2.3. Instruments

The target scale for the validation, in this case, was the MAL-30, which is a scale based on a semi-structured interview that assesses the quantity and quality of the use of the paretic upper limbs in the performance of ADLs and IADLs in post-stroke individuals. It contains 30 items in each of the quantity and quality subscales, and the scores are provided for each of them independently, assigning a score ranging from 0 (never uses the affected arm to perform the activity) to 5 (ability to use the affected arm for this activity as effectively as the time before the stroke). All the scores are summed, and the total score is obtained from the average of the items answered (it is not necessary to answer all the items, but only those for which the affected arm was used before the stroke). Higher scores on this scale express both a higher quantity and quality of movement and a normalized use of the affected upper limb in the performance of activities. The measure is based on the patient’s self-report and not on the direct assessment of their motor function. Several studies have demonstrated the good psychometric properties of the instrument [17,20,22,30,37] in populations other than the Spanish population.

The FMA-EU was used as the gold standard for the criterion validity, since it has proven to be a scale that is widely used in clinical practice due to its good psychometric properties and has been validated [9,38] for the Spanish population. The FMA-UE is an instrument used to measure the functionality of the paretic upper limb in post-stroke patients. The scale contains 33 items with a total of 66 points, rated from 0 to 2 (0, no realization; 2, complete realization) [39,40].

For convergent validity, several scales were used to measure, on the one hand, the functionality of the affected upper limb and the level of independence in the performance of ADLs and IADLs, and on the other hand, the QoL.

The following scales were used for measuring the functionality and the level of independence:

- ARAT is a tool that measures the patient’s ability to use the upper limb to perform specific daily tasks involving grasping, pressing or gross motor skills. It consists of 19 items with a total score of 57, each item being rated from 0 to 3 (0, no performance; 3, normal performance). The inability to perform the different items of the scale can be a reliable indicator of the limited ability to perform the activity with the affected upper limb. It is a valid and reliable instrument for people with stroke [41,42].
- The FIM-FAM scale is an instrument used as a global measure of disability to determine the degree of independence a person has in his or her ADLs. The FIM can be used independently (18 items) or in conjunction with the FAM (+12 items), with a scoring system of 1 (total dependence) to 7 (total independence), with a total of 210 [42,43].
- The Lawton and Brody index assesses the ability to perform the IADLs by means of 8 items that are rated between 0 and 1, with 0 indicating no independent performance of the different activities [44,45].
- Finally, the BBT is a simple outcome measure used to assess gross motor skills in stroke patients. The battery consists of 150 wooden blocks that must be moved between the two holes of the box (separated by a partition) over 1 min. The scoring consists of manually counting the number of blocks moved [46,47].

QoL measuring tool:

- The ECVI-38 scale is a questionnaire that specifically measures the QOL of stroke patients. It contains 38 items distributed in 8 domains: the physical state, communication, cognition, emotions, feelings, ADLs, common activities of daily living and socio-familial functions. It is scored from 1 (no difficulty) to 5 (extreme difficulty). The higher the score is, the lower the QoL is. The Spanish version has demonstrated a good validity, reliability and feasibility [25,48].

### 2.4. Data Analysis

A psychometric analysis using the MAL-30 scale was carried out by performing a reliability analysis and a validity analysis.

On the one hand, for the reliability analysis, the internal consistency was tested by means of Cronbach’s alpha, correlations between the items, the item–total correlation and the half-and-half test.

On the other hand, for the validity analysis, the criterion validity was tested. For this purpose, it was compared with the 4 subscales of which the FMA-UE scale is comprised (considering the FMA scale as the gold standard [49]), and the convergent validity was assessed by correlation with the ARAT, box and blocks, FIM-FAM, Lawton and Brody scale and ECVI-38.

## 3. Results

The sample consisted of 223 persons who had previously experienced a stroke. The mean age of the sample corresponded to 61.81 years (SD ± 11.54), with 44.4% of the patients being men and 55.6% being women. In total, 57.1% were married or in a couple, followed by those who were separated or divorced (20%), single (14.3%) and widowed (8.6%). Of the participants, 88.6% had children and 64.7% also had a basic education.

The most prevalent type of stroke was ischemic, with 87.1%, followed by hemorrhagic (9.7%) and transitory ischemic stroke (TIS) (3.2%). The etiology was mostly hemorrhagic (35.5%), although there were also other types, such as atherothrombotic (29%), cardioembolic (9.7%) and small vessel disease (9.7%), and the rest (16.1%) were due to rare or undetermined causes. Likewise, most of the participants had no family history of stroke (74.2%) and had not had a previous stroke (87.1%). In addition, 55.2% had right hemisphere and 44.8% had left hemisphere involvement, and 85 (37.9%) people had their dominant upper limb affected, while 106 (47,3%) had their non-dominant upper limb affected.

### 3.1. Reliability Analysis

#### Internal Consistency

- Cronbach’s alpha

First, the reliability was analyzed using Cronbach’s alpha to verify the internal consistency of the instrument. In addition, the item–total correlation, the squared correlation (variance explained) with the items of the scale and the value of Cronbach’s alpha when each item was eliminated were analyzed.

Since this is a scale in which the same items are measured for the quantity and quality and which offers a total score for each, but not a total score that unifies both, it was necessary to analyze Cronbach’s alpha for the quantity and quality items separately.

For the quantity, an α = 0.990 was obtained. Cronbach’s α for each of the deleted items ranged from 0.989–0.990, and the total corrected item correlation was greater than 0.497 in all cases.

For the quality, an α = 0.991 was obtained. The α for each of the deleted items ranged from 0.991–0.995, and the total correlation of corrected items was greater than 0.504.

- Correlations between the items that form the scale and item–total correlations

In all cases, high correlations appeared between each of the items that form the MAL-30, as did correlations with the total score of the tools for the quantity and quality, assessed separately. For the quantity, the correlation coefficients ranged from 0.305 to 0.949 (*p* < 0.001), and for the quality, the coefficients were between 0.426 and 0.964 (*p* < 0.001). The total score for the quantity also correlated with that for the quality (*p* < 0.001).

- Half-and-half test

The reliability of the MAL-30 instrument was also examined through the half-and-half test for the quantity and quality, respectively, as shown in Table 1 and Table 2, with a Spearman–Brown value (equal length) indicating a very good reliability.

### 3.2. Validity Analysis

#### 3.2.1. Criterion Validity

The total MAL-30 quantity and quality scores correlated positively with the scores for the three dimensions and the total score for the FMA-EU scale, chosen as the gold standard for comparison, so that the higher the MAL-30 score was, the higher the scores on the FMA-EU scale were, except in the case of the MAL-30 quantity score with respect to the sensitivity dimension of the FMA-EU, in which there was no significant correlation (Table 3).

#### 3.2.2. Convergent Validity

Table 4 and Table 5 show the correlations of the MAL-30 total quantity and quality scores with the ARAT instrument (left and right), BBT (left and right), FIM-FAM, Lawton and Brody scale and ECVI-38 scores.

The correlations with the ARAT, BBT, FIM-FAM and Lawton and Brody scales were found to be significant. This means that when there is a worse upper limb functionality, the performance of ADLs will be worse. The correlations with ECVI-38 were significantly negative, so that when the upper limb function is worse, the QoL will also be worse.

## 4. Discussion

The aim of the study was to validate the MAL-30 assessment instrument for post-stroke patients in the Spanish population. The validation of an assessment instrument is of great necessity in order to guarantee its reliability and validity, and, in addition, its use offers evidence of the results. The validation process is first carried out in the original language, and then the existing versions are validated when translations and/or modifications are developed [50].

The content validation processes that are usually used require the assessment of the items of the scale by a panel of experts. However, in the case of the MAL-30 scale, it corresponds to an instrument that is widely used in clinical practice; thus, it requires the support of the professionals who use it. Likewise, the translation of this instrument has been used in other studies [20,31,32,33,34]. This content validation process can therefore be considered as already completed for this assessment tool.

Our results show that the Cronbach’s alpha coefficient obtained was 0.990 for the quantity subscale and 0.991 for the quality subscale, indicating a very good internal consistency of the tool, as it was higher than 0.8. For each of the items removed, Cronbach’s alpha ranged between 0.989 and 0.990 for the quantity subscale and between 0.991 and 0.995 for the quality subscale. Additionally, the total correction of the corrected items was above 0.42 in all cases, which means that none of the items needed to be deleted from the instrument. It also showed a good homogeneity of the statements of which it is composed, indicated by both the total score and the correlation between the items, and, in principle, all the items measure the same construct [39]. A review of 55 studies analyzing the internal consistency of the MAL-30 found that, for this scale, Cronbach’s alpha was 0.94 for both scales when applied to stroke patients [30].

The good internal consistency of the MAL-30 was also confirmed by a Spearman–Brown coefficient score above 0.080, which was obtained through the half-and-half test. Thus, the high internal consistency demonstrated by the MAL-30 ensures the homogeneity of its items, guaranteeing a linear relationship between the sum of the percentages of the items measuring the mean construct and those measuring a single construct [39]

The MAL-30 scale score and its quantity and quality subscales correlated positively with the ARAT, BBT, FIM-FAM and Lawton and Brody index and negatively with the ECVI-38 scores. This means that when the person has a lower use in terms of quantity and a worse quality of movement of the upper limb in the performance of ADLs and IADLs, worse functionality scores and, therefore, greater limitations in the performance of ADLs and a worse QoL will be observed. Previous research has proven the association between the MAL-30 and functionality and ADL performance. Therefore, the results indicated that the MAL-30 showed a good convergent validity for the sample. The systematic review by Amaral Saliba et al. [30] confirmed that the existing versions of the MAL scale have adequate psychometric properties for measuring the quantity and quality of the use of the affected upper limb in ADLs among post-stroke patients. Another study obtained good results in terms of the clinical application of the MAL, identifying its great potential for the assessment of the upper limb in patients with chronic-stroke-related hemiplegia [27].

It is necessary to take into account the limitations of the article in terms of the results of the research, such as the non-probabilistic sampling. The limitations of the self-report measures may have resulted in the collection of erroneous information that may have led to misleading results and conclusions or type-I research errors. However, such instruments are commonly used, and their psychometric properties should also be studied.

On the other hand, we can also highlight, as strengths of the study, the fact that it was a multicenter study that addressed the needs and characteristics of a specific population, which also required a validation assessment of the instrument. Through the results of this study, we can recommend the use of the MAL-30 scale for the assessment of the use of the affected upper limb in the ADL and IADL of post-stroke patients in Spain. In addition, the study was conducted using a blinded procedure, since the people in charge of the statistical analysis were different from those in charge of the data collection.

## 5. Conclusions

The Spanish version of the MAL-30 scale has proved to be a tool with a good reliability and validity that can be used to measure the use, in terms of quantity and quality, of the affected upper limb in post-stroke individuals in a Spanish sample.

The internal consistency shows the good reliability of MAL-30, as well as its good criterion validity when compared with the FMA-EU, and its convergent validity is also appropriate.

Therefore, the use of the MAL-30 tool can be recommended as part of the assessment and as a step prior to the rehabilitation of patients who have experienced a stroke, with the aim of planning an individualized and personalized treatment adapted to the characteristics and needs of each patient. It is also essential to evaluate the monitoring and effectiveness of the program implemented, verifying the results obtained through it.

## Figures and Tables

**Table 1:** Half-and-half method reliability statistics: quantity.

Cronbach’s alpha	Part 1	Value	0.988
		N of elements	15 ^a^
	Part 2	Value	0.973
		N of elements	15 ^b^
	Total N of elements		30
Correlation between forms			0.974
Spearman–Brown’s coefficient	Equal length		0.987
	Non equal length		0.987
Guttman coefficient of the two halves			0.987

^a^. The elements are as follows: 1. Turn on the light with a switch QUANTITY, 2. Open a chest of drawers QUANTITY, 3. Take clothes out of drawer QUANTITY, 4. Pick up the phone QUANTITY, 5. Clean a surface with a cloth QUANTITY, 6. Get out of the car QUANTITY, 7. Open a refrigerator QUANTITY, 8. Open the door by turning the handle QUANTITY, 9. Use the TV remote QUANTITY, 10. Wash hands (includes applying soap, does not include turning on the faucet) QUANTITY, 11. Open and close the water faucet QUANTITY, 12. Dry hands QUANTITY, 13. Put on socks QUANTITY, 14. Take off socks QUANTITY, 15. Put on shoes (including laces) QUANTITY. ^b^. The elements are as follows: 16. Remove shoes (including laces) QUANTITY, 17. Get up from chair with armrests QUANTITY, 18. Pull the chair from the table to sit QUANTITY, 19. Push a chair towards the table after sitting QUANTITY, 20. Grab a glass, bottle, or cup to drink QUANTITY, 21. Brushing teeth (does not include applying the paste) QUANTITY, 22. Apply makeup, cream QUANTITY, 23. Use key to open door QUANTITY, 24. Writing on paper QUANTITY, 25. Carrying an object in hand QUANTITY, 26. Use fork or spoon to eat QUANTITY, 27. Combing hair QUANTITY, 28. Take a cup by the handle QUANTITY, 29. Button a shirt QUANTITY, 30. Eat a bread or sandwich QUANTITY.

**Table 2:** Half-and-half method reliability statistics: quality.

Cronbach’s alpha	Part 1	Value	0.978
		N of elements	15 ^a^
	Part 2	Value	0.989
		N of elements	15 ^b^
	Total N of elements		30
Correlation between forms			0.978
Spearman–Brown’s coefficient	Equal length		0.998
	Non equal length		0.989
Guttman coefficient of two halves			0.989

^a^. The elements are: 1. Turn on the light with a switch QUALITY, 2. Open a chest of drawers QUALITY, 3. Take clothes out of drawer QUALITY, 4. Pick up the phone QUALITY, 5. Clean a surface with a cloth QUALITY, 6. Get out of the car QUALITY, 7. Open a refrigerator QUALITY, 8. Open the door by turning the handle QUALITY, 9. Use the TV remote QUALITY, 10. Wash hands (includes applying soap, does not include opening the faucet) QUALITY, 11. Open and close the water faucet QUALITY, 12. Drying hands QUALITY, 13. Put on socks QUALITY, 14. Take off socks QUALITY, 15. Put on shoes (includes laces) QUALITY. ^b^. The elements are: 16. Take off shoes (including laces) QUALITY, 17. Get up from a chair with armrests QUALITY, 18. Pull the chair from the table to sit QUALITY, 19. Push a chair to the table after sitting QUALITY, 20. Grab a glass, bottle or cup to drink QUALITY, 21. Brushing teeth (does not include applying paste) QUALITY, 22. Apply makeup, cream QUALITY, 23. Use key to open door QUALITY, 24. Writing on paper QUALITY, 25. Carry an object in hand QUALITY, 26. Use fork or spoon to eat QUALITY, 27. Combing hair QUALITY, 28. Take a cup by the handle QUALITY, 29. Button a shirt QUALITY, 30. Eat a bread or sandwich QUALITY.

**Table 3:** Pearson’s correlation MAL30—FMA-UE.

		Motor Function FMA-UE	Sensibility FMA-UE	Pain Range FMA-UE	Total FMA-UE
Average MAL-30 quantity	Correlation coefficient	0.320 **	0.123	0.213 **	0.322 **
	Sig. (bilateral)	<0.001	0.066	<0.001	<0.001
	N	223	223	223	223
Average MAL-30 quality	Correlation coefficient	0.803 **	0.489 **	0.443 **	0.805 **
	Sig. (bilateral)	<0.001	<0.001	<0.001	<0.001
	N	223	223	223	223

FMA-UE: Fugl-Meyer assessment for the upper extremity; MAL-30: motor activity log. ** The correlation is significant at the 0.01 level (bilateral).

**Table 4:** Pearson’s correlation: MAL-30–ARAT, BBT.

		ARAT (Left)	ARAT (Right)	BBT (Left)	BBT (Right)
Average MAL-30 quantity	Correlation coefficient	0.173 **	0.227 **	0.187 **	0.294 **
	Sig. (bilateral)	0.010	0.001	0.005	<0.001
	N	223	223	223	223
Average MAL-30 quality	Correlation coefficient	0.574 **	0.473 **	0.587 **	0.496 **
	Sig. (bilateral)	<0.001	<0.001	<0.001	<0.001
	N	223	223	223	223

ARAT: action research arm test; BBT: box and blocks test; MAL-30: motor activity log. ** The correlation is significant at the 0.01 level (bilateral).

**Table 5:** Pearson’s correlation: MAL-30–FIM-FAM, Lawton and Brody index and ECVI-38.

		FIM-FAM	Lawton y Brody	ECVI-38
Average MAL-30 quantity	Correlation coefficient	0.353 **	0.272 **	−0.320 **
	Sig. (bilateral)	<0.001	<0.001	<0.001
	N	223	223	223
Average MAL-30 quality	Correlation coefficient	0.766 **	0.690 **	−0.735 **
	Sig. (bilateral)	<0.001	<0.001	<0.001
	N	223	223	223

FIM-FAM: functional independence measure–functional assessment measure; ECVI-38: Escala Calidad de Vida Ictus (stroke quality of life scale); MAL-30: motor activity log. ** The correlation is significant at the 0.01 level (bilateral).
